# Impact of training data composition on the generalizability of convolutional neural network aortic cross-section segmentation in four-dimensional magnetic resonance flow imaging

**DOI:** 10.1016/j.jocmr.2024.101081

**Published:** 2024-08-08

**Authors:** Chiara Manini, Markus Hüllebrand, Lars Walczak, Sarah Nordmeyer, Lina Jarmatz, Titus Kuehne, Heiko Stern, Christian Meierhofer, Andreas Harloff, Jennifer Erley, Sebastian Kelle, Peter Bannas, Ralf Felix Trauzeddel, Jeanette Schulz-Menger, Anja Hennemuth

**Affiliations:** a Deutsches Herzzentrum der Charité (DHZC), Institute of Computer-assisted Cardiovascular Medicine, Berlin, Germany; b Charité – Universitätsmedizin Berlin, corporate member of Freie Universität Berlin and Humboldt Universität zu Berlin, Berlin, Germany; c Fraunhofer MEVIS, Berlin, Germany; d Department of Diagnostic and Interventional Radiology, Tübingen University Hospital, Tübingen, Germany; e German Center for Cardiovascular Research (DZHK), Berlin, Germany; f Congenital Heart Disease and Pediatric Cardiology, German Heart Center Munich, Munich, Germany; g Department of Neurology and Neurophysiology, University Medical Center Freiburg - Faculty of Medicine, University of Freiburg, Freiburg, Germany; h Department of Cardiology, Angiology and Intensive Care Medicine, Deutsches Herzzentrum der Charité - Universitätsmedizin Berlin, corporate member of Freie Universität Berlin and Humboldt Universität zu Berlin, Berlin, Germany; i Department of Diagnostic and Interventional Radiology and Nuclear Medicine, University Medical Center Hamburg-Eppendorf, Hamburg, Germany; j Charité – Universitätsmedizin Berlin, corporate member of Freie Universität Berlin and Humboldt-Universität zu Berlin, ECRC Experimental and Clinical Research Center, Lindenberger Weg 80, 13125 Berlin, Germany; k Working Group on Cardiovascular Magnetic Resonance, Experimental and Clinical Research Center, a joint cooperation between the Charité – Universitätsmedizin Berlin and the Max-Delbrück-Center for Molecular Medicine, Berlin, Germany; l Charité – Universitätsmedizin Berlin, corporate member of Freie Universität Berlin and Humboldt-Universität zu Berlin, Department of Anesthesiology and Intensive Care Medicine, Charité Campus Benjamin Franklin, Hindenburgdamm 30, 12203 Berlin, Germany; m Department of Cardiology and Nephrology, Helios Hospital Berlin-Buch, Berlin, Germany

**Keywords:** Deep learning, 4D flow MRI, Thoracic aorta, BAV, Segmentation

## Abstract

**Background:**

Four-dimensional cardiovascular magnetic resonance flow imaging (4D flow CMR) plays an important role in assessing cardiovascular diseases. However, the manual or semi-automatic segmentation of aortic vessel boundaries in 4D flow data introduces variability and limits the reproducibility of aortic hemodynamics visualization and quantitative flow-related parameter computation. This paper explores the potential of deep learning to improve 4D flow CMR segmentation by developing models for automatic segmentation and analyzes the impact of the training data on the generalization of the model across different sites, scanner vendors, sequences, and pathologies.

**Methods:**

The study population consists of 260 4D flow CMR datasets, including subjects without known aortic pathology, healthy volunteers, and patients with bicuspid aortic valve (BAV) examined at different hospitals. The dataset was split to train segmentation models on subsets with different representations of characteristics, such as pathology, gender, age, scanner model, vendor, and field strength. An enhanced three-dimensional U-net convolutional neural network (CNN) architecture with residual units was trained for time-resolved two-dimensional aortic cross-sectional segmentation. Model performance was evaluated using Dice score, Hausdorff distance, and average symmetric surface distance on test data, datasets with characteristics not represented in the training set (model-specific), and an overall evaluation set. Standard diagnostic flow parameters were computed and compared with manual segmentation results using Bland-Altman analysis and interclass correlation.

**Results:**

The representation of technical factors, such as scanner vendor and field strength, in the training dataset had the strongest influence on the overall segmentation performance. Age had a greater impact than gender. Models solely trained on BAV patients’ datasets performed well on datasets of healthy subjects but not vice versa.

**Conclusion:**

This study highlights the importance of considering a heterogeneous dataset for the training of widely applicable automatic CNN segmentations in 4D flow CMR, with a particular focus on the inclusion of different pathologies and technical aspects of data acquisition.

## Graphical abstract

**Figure:**
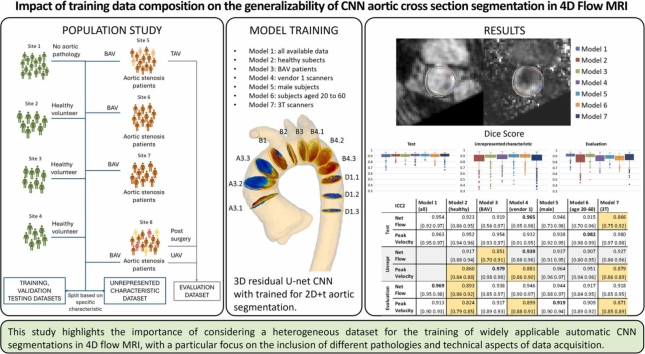

## 1 Background

Four-dimensional cardiovascular magnetic resonance flow imaging (4D flow CMR) enables a comprehensive visualization and evaluation of aortic hemodynamics [1] that can support the assessment and understanding of cardiovascular diseases [2], [3], [4], [5], [6]. For application in clinical practice, standardized post-processing procedures and quantification concepts have been jointly proposed by research experts and clinicians [7], [8]. They suggest the assessment of hemodynamic properties based on two-dimensional (2D) segmentations of aortic cross-sections in all timeframes of the 4D flow CMR image series.

Previous studies analyzed the reproducibility of the corresponding hemodynamic parameters in healthy volunteers showing their dependency on the segmentations [9], [10], [11], [12], [13]. They found that the variability by expert segmentations was higher than between scans and reported the impact on extracted flow parameters. Vessel wall parameters, such as wall shear stress (WSS) [9], [10], [12], were strongly influenced by segmentation variation, and the strongest effect was observed in the ascending aorta (AAo) [11]. Zimmermann et al. [13] demonstrated the effects of slight changes in vessel contours on the resulting WSS. They found a strong increase in systolic values when shrinking contours so that they were placed inside the vessel lumen. Juffermans et al. [14] found increased differences in expert segmentations in patient datasets compared to healthy controls. Casciaro et al. [15] also reported positive and negative blood flow to be significantly affected by segmentation variation, more evidently in the AAo and in subjects with bicuspid aortic valve (BAV). Huellebrand et al. [16] further reported considerable inter-scanner differences for flow parameters as well as radiomics features in the AAo of volunteers.

A variety of software solutions for post-processing and analysis of 4D flow CMR offer semi-automatic aortic vessel segmentation to reduce observer-based variation. The temporally resolved cross-sectional segmentations required for the extraction of clinically relevant flow parameters can either be derived from a 4D (three-dimensional [3D]+t) aorta segmentation or from a two-dimensional (2D)+t segmentation of a multiplanar reformation (MPR) that can be defined as a cross-section of a 3D aorta segmentation [7].

Atlas-based approaches can generate fully automatic 3D segmentations and propagate them through the cardiac cycle to provide 4D (3D+t) segmentation masks [17], but they are computationally expensive, relatively slow, and limited with respect to fitting individual anatomic shapes.

Deep learning (DL) has shown the potential to enable fast automatic and reproducible 4D flow CMR segmentation and analysis [18]. Segmentation solutions in 3D [19] and 3D with each frame as independent sample [20], [21] achieve good results for 4D flow data, comparable to expert annotation. However, published models are typically trained on datasets with specific imaging sequences and scanner manufacturers [19], [20], [21], [22].

2D+t cross-sectional vessel segmentations can be performed semi-automatically based on a manual segmentation of a single timeframe, which is then automatically propagated to the full time series [16], [23], [24]. Successful convolutional neural network (CNN)-based approaches for aortic cross-section 2D segmentation have been published for 2D flow CMR sequences [25]. Fujiwara et al. [26] proposed a 3D DL approach that further addressed the problem of variations in imaging sequences and scanner manufacturers [27], [28], [29], expanding a previously developed CNN [19] by using pediatric data from two centers. The model performance was comparable to manual annotations and CNNs trained on single-site data. The two combined datasets differ in site, scanner model, vendor, and magnetic field strength, so that the impact of the single characteristics on the model generalizability could not be analyzed independently.

Automatic accurate reproducible segmentations could broaden the applicability of 4D flow CMR in clinical settings; the development of a DL model that is widely applicable across different sites, vendors, sequence types, and pathologies, including their anatomical variability might solve this task.

The goal of this work is to assess the generalizability of a DL segmentation model on multi-site, multi-scanner, and multi-sequence 4D flow CMR data and investigate how the representation of technical imaging properties (site, scanner vendor and model, field strength) or subject characteristics (pathology, age, and gender) in the training data influence the segmentation and quantification performance.

To this end, we trained a state-of-the-art CNN on subsets of heterogeneous multi-site and multi-vendor data from eight different sites leaving out one technical property or patient characteristic per training set.

The outputs of the CNN segmentation models were evaluated via comparison with the manual ground truth annotation and the effect of segmentation differences on derived clinical parameters such as through-plane flow (net flow) and maximum velocity.

## 2 Methods

We considered data from eight scanning sites to obtain a heterogeneous multi-vendor dataset. We trained seven models, each neglecting a specific subject or scanner characteristic. Based on the filtering criteria applied to the training data, two subsets per model emerged for the model performance assessment: one in which the characteristic feature representation corresponds to the training set (test set) and another containing datasets with the neglected characteristic (unrepresented characteristic set). To generate unbiased subsets of the available data for training, testing, and evaluation of models representing different characteristics, we ensured that data from the same subject did not appear in different sets. To further assess the generalizability and compare the models, we created an overall evaluation set, which contains datasets with additional aortic valve configurations and post-treatment datasets.

### 2.1 Study population

Two hunderd sixty 4D flow MRI acquisitions of different subjects were retrospectively included in this study:

• 131 subjects without known aortic pathology [4].
• 23 healthy volunteers were scanned at three different sites [30], [31].
• 106 bicuspid aortic valve patients were scanned at four different sites [32], [33], [34], [35].

**Fig. 1:**
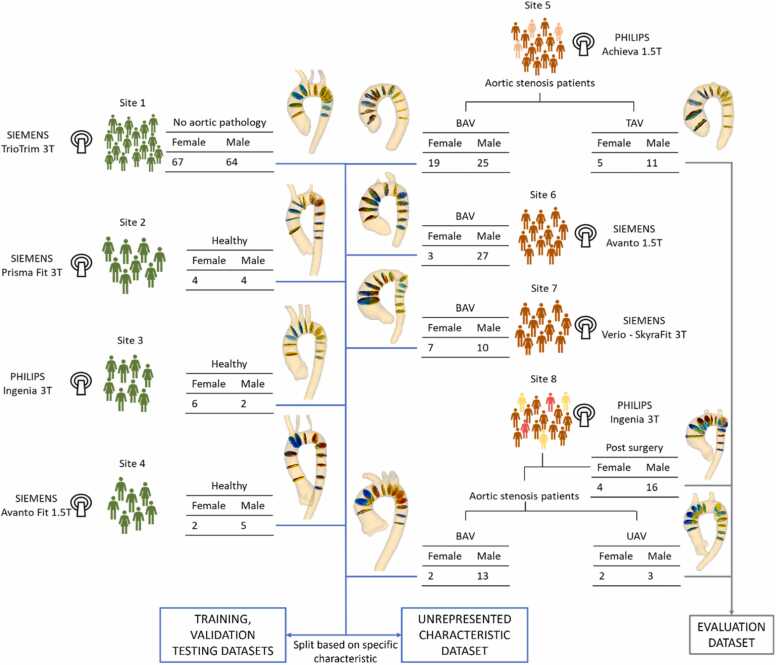
Flowchart of the data subset generation. Image data from patients with no aortic pathology (site 1), healthy volunteers (sites 2–4), and bicuspid aortic valve (BAV) patients (sites 5–8) are used to train and test the proposed automatic aortic cross-section segmentation models. Evaluation dataset: stenotic tricuspid aortic valve (TAV) patients from site 5, stenotic unicuspid aortic valve (UAV) patients from site 8, and postoperative scanners from site 8 have been separated to be used for additional comparative evaluation of the models

Detailed subject and scanner information is provided in Table 1 and Fig. 1.

**Table 1:** Properties of the main dataset used for training and testing of the CNN models.

	Population study	Healthy volunteers			BAV			
	Site 1 [4]	Site 2 [30]	Site 3 [30]	Site 4 [31]	Site 5 [34]	Site 6 [32]	Site 7 [35]	Site 8 [33]
Subjects	131	8	8	7	44	30	17	15
Cross-sections	1536	95	95	82	518	346	159	45
Age (years)	50 ± 16	27 ± 8	25 ± 5	28 ± 8	64 ± 19	29 ± 11	60 ± 24	43 ± 12
Female	67 (51%)	4 (50%)	6 (75%)	2 (29%)	19 (43%)	3 (10%)	7 (41%)	2 (13%)
Scanner	Siemens TrioTim	Siemens Prisma Fit	Philips Ingenia	Siemens Avanto Fit	Philips Achieva	Siemens Avanto	Siemens Verio-SkyraFit	Philips Ingenia
Field strength	3T	3T	3T	1.5T	1.5T	1.5T	3T	3T
Venc (cm/s)	150	150	250	150	350−600	150−400	150−250	200
ECG gating	Prospective	Prospective	Retrospective	Prospective	Retrospective	Prospective	Prospective	Prospective
Echo time (ms)	2.54	2.6	2.2	2.4	2.2	2.44	2.6	2.02
Flip angle (°)	7	8	5	8	7	8	7−9	8
Voxel size (mm^3^)	2.5 × 2.1 × 2.5	2.7 × 2.3 × 2.6	2.8 × 2.8 × 2.8	2.25 × 2.25 × 2.5	2.5 × 2.5 × 2.5	2.5 × 2.5 × 2.5	2.7 × 2.3 × 2.6	2.5 × 2.5 × 2.5
Timeframes/cycle	40 ± 6	20	25	20	25	25	20 ± 2	24
Temporal resolution (ms)	20	40.8	28	40.64	38	40	40.8	24−38

*CNN* convolutional neural network*, BAV* bicuspid aortic valve*, Venc* velocity encoding*, ECG* electrocardiogram

We included volunteers and BAV patients in the main dataset for model training and testing (Table 1). The overall evaluation set contains the following additional aortic valve configurations and post-surgical datasets:

• 16 patients (190 cross-sections) with stenotic tricuspid aortic valve (TAV) from site 5.
• 5 patients (57 cross-sections) with stenotic unicuspidal aortic valve from the dataset of site 8.
• 20 post‐surgery scans (220 cross-sections) from site 8.

**Fig. 2:**
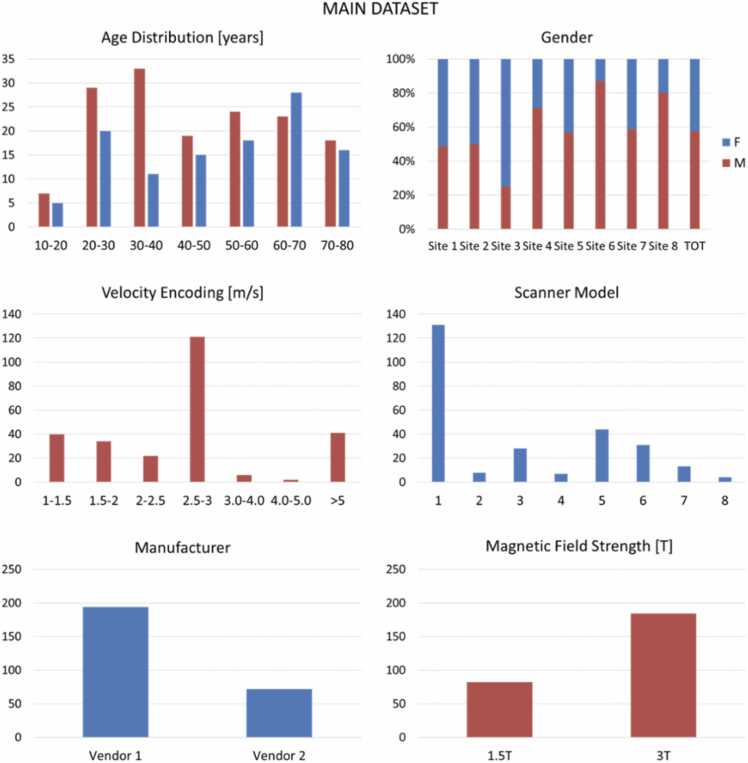
Properties of the main dataset

**Fig. 3:**
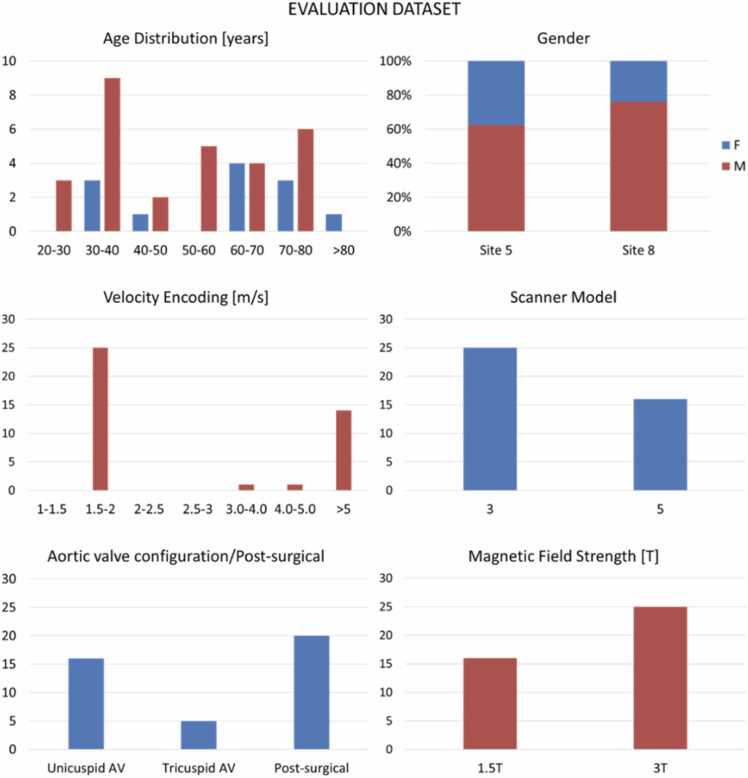
The overall evaluation dataset consists of 16 stenotic tricuspid aortic valve patients, 5 unicuspid stenotic aortic valves, and 20 post-treatment scans. *AV* aortic valve

Fig. 1 provides an overview of the data and the different subsets, detailed statistical information is shown in Figs. 2 and 3.

### 2.2 Data processing and annotation

4D flow CMR images were processed and annotated using MEVISFlow (Fraunhofer Institute for Digital Medicine MEVIS, software version 11.5, Bremen, Germany) [24]. Preprocessing included background phase offset correction with a polynomial fit and phase unwrapping using the PRELUDE algorithm [36]. The aortic centerline was derived from the 3D aorta segmentation of the phase-contrast magnetic resonance angiography.

**Fig. 4:**
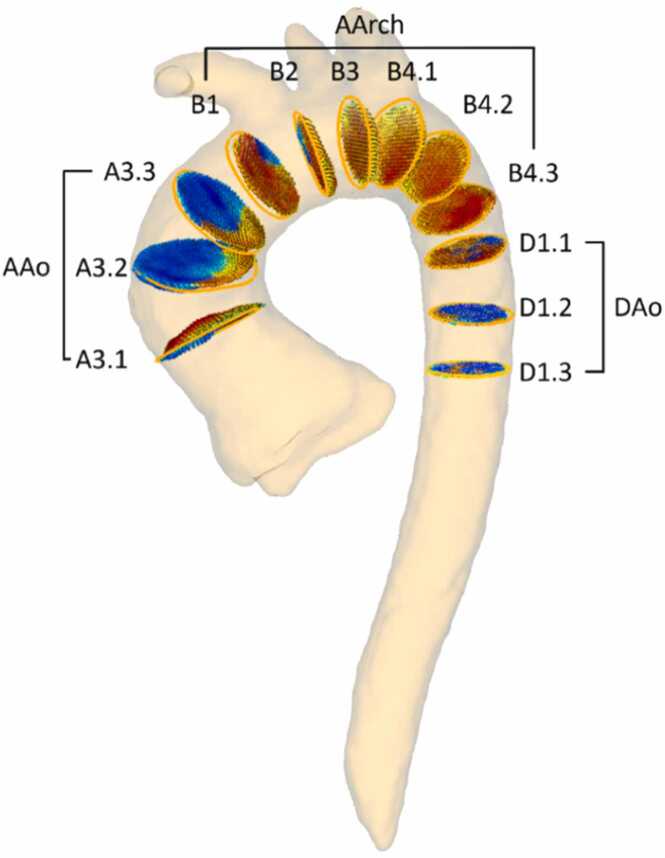
Twelve cross-sectional planes covering the ascending aorta (AAo), the aortic arch (AArch), and the descending aorta (DAo). A3.1, B1, B2, B3, B4.1, and D1.1 were placed manually by expert users. Based on these positions A3.2–3, B4.2–3, D1.2–3 are automatically placed with equidistant spacing

Following the protocol defined by Schafstedde et al. [23], four cross-sectional planes were placed perpendicular to the aortic centerline in specific locations (Fig. 4): distal to the coronary ostia (A3.1), proximal to the brachiocephalic trunk (B1), between the branches (B2 and B3), distal to the subclavian artery (B4.1), and adjacent to the pulmonary artery (D1.1). The additional cross-sections were automatically placed with equal distance, resulting in a total number of 2981 cross-sections for the main set and 467 for the overall evaluation set.

Three experts manually annotated the vessel contour on all timeframes (15 to 57 frames/cardiac cycle) of each cross-section considering magnitude and velocity images after an initial training. Each user segmented cases from 2 or 3 sites. Segmentations were randomly cross-checked by another user.

Detailed information about the datasets can be found in the corresponding publications [4], [30], [31], [32], [33], [34], [35].

### 2.3 Training setup

Seven data subsets resulting from selecting different technical properties or patient characteristics were randomly divided into 80% for training, 10% for validation, and 10% for testing. Splits were performed site-wise to maintain the proportion between the different scanning sites and subject-wise to avoid data leakage and biases in validation and testing [29].

The subsets of the main dataset (Fig. 2) were chosen as follows:

• Model 1: All available data (N_train_ = 202, N_val_ = 28, N_test_ = 30)
• Model 2: Only healthy subjects (N_train_ = 121, N_val_ = 16, N_test_ = 17)
• Model 3: Only BAV patients (N_train_ = 82, N_val_ = 11, N_test_ = 13)
• Model 4: Only vendor 1 scanners (N_train_ = 150, N_val_ = 21, N_test_ = 22)
• Model 5: Only male subjects (N_train_ = 113, N_val_ = 17, N_test_ = 20)
• Model 6: Subjects aged 20 to 60 (N_train_ = 124, N_val_ = 17, N_test_ = 20)
• Model 7: Only 3T scanners (N_train_ = 141, N_val_ = 18, N_test_ = 20)

The datasets left out in the training of the respective models were used to assess the model performance on data with unseen characteristics. The evaluation dataset was composed to enable the comparative assessment of the models’ performance and generalizability on an unrepresented aortic valve configuration and post-surgical data. Detailed statistical information for each model-specific split is provided in the Supplementary Material.

### 2.4 Data preprocessing for model training and application

Per cross-section annotation, we generated four corresponding 2D+t MPR sequences representing the magnitude and the three velocity components as well as one corresponding mask sequence. Z-score normalization was applied to the magnitude sequence. To ensure an in-plane resolution of 1.54 mm^2^ and a temporal resolution of 64 timesteps per sequence, a spatiotemporal resampling with bilinear interpolation was applied for the two spatial directions, and nearest-neighbor interpolation was used for the temporal interpolation. A final in-plane dimension of 64 × 64 was obtained via padding or cropping. The full inclusion of the vessel cross-section was ensured using centerline-based plane positioning and a random weighted cropping transformation that used the mask information to locate the crop region. We applied basic data augmentation including translation, rotation, and flipping (cross-section normal). Expert segmentation masks were resampled spatiotemporally with nearest-neighbor interpolation. The magnitude and velocity 2D+t sequences were concatenated as channels, resulting in the final four channels input for the model.

### 2.5 Model architecture

We chose an enhanced version of a 3D U-net with residual units that uses convolutions to change the input dimension to match the output [37]. Each of the six layers has a skip connection between the encode and decode path. Downsampling and upsampling operations are performed at the beginning of each block via strided convolutions and strided transpose convolutions with stride = 2, respectively. Inference is performed with a sliding window with an overlap of 0.5. We trained the model to generate 2D+t aortic cross-sectional segmentations (x, y, t). A sigmoid function generates per voxel probabilities to belong to the vessel cross-section, and a threshold of 0.5 was set to generate a binary mask. Unconnected areas assumed to represent noise and artifacts are removed by keeping only the largest connected component of the resulting mask. A composite loss function (cross entropy and Dice loss) was used during training. The model was implemented in Python 3.7.6 (Python Software Foundation, Beaverton, Oregon) using monai 0.8.1 [38]. Training and testing were performed on an AMD EPYC 7302 16-Core Processor with a Nvidia A40 graphics processing unot (GPU).

### 2.6 Model evaluation and statistical analysis

The segmentation results generated by the seven models on the overall evaluation set and their corresponding test and unrepresented characteristic sets were compared with the expert segmentation masks using the Dice score (DS), Hausdorff distance (HD), and average symmetric surface distance (ASSD) (computed with monai 0.8.1, see Supplementary Material for details).

The model-generated segmentation masks were imported into MEVISFlow to calculate through-plane flow (net flow), cross-section area, and peak velocity to enable a comparison with the corresponding clinical parameters derived from the expert segmentations. To evaluate the agreement of the hemodynamic parameters, we computed interclass correlation coefficients (ICC2, two-way random, single measure, absolute agreement) and confidence intervals. Additionally, Bland-Altman analysis was performed, and limits of agreement were reported together with bias. The analysis was performed with pingouin 0.5.3.

### 2.7 Intra- and inter-observer variability analysis

The experts re-segmented 10 cases (out of the 30 in the test set from model 1, selected maintaining site proportions) to enable the assessment of intra- and inter-observer variability. We computed ICC2, confidence intervals, bias, and limits of agreement (Bland-Altman analysis) for both net flow and peak velocity in the intra- and inter-observer analysis.

## 3 Results

**Fig. 5:**
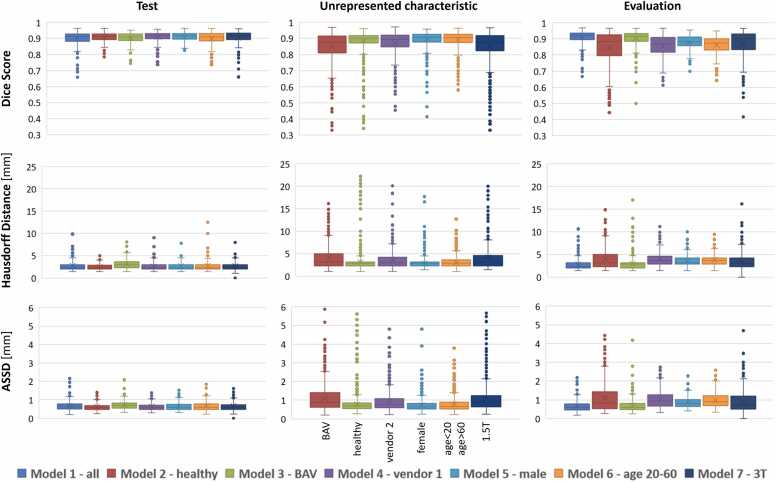
Box plots of the evaluation metrics computed on the model-specific test sets and unrepresented sets, as well as on the common evaluation set. Minimum values, percentiles (25th, 50th, and 75th), mean, maximum and outlier values are shown. *ASSD* average symmetric surface distance

The seven segmentation models were successfully trained using the respective training and validation sets. The resulting models were applied to their test and unrepresented characteristics sets (N_model2_ = 106, N_model3_ = 154, N_model4_ = 67, N_model5_ = 110, N_model6_ = 99, and N_model7_ = 81), as well as the common overall evaluation set (N = 41) to generate the 2D+t segmentations of the aortic vessel cross-sections. Inference time of the models on the evaluation set was 13 to 18 ms per model with an AMD EPYC 7302 16-Core Processor with a Nvidia A40 GPU. The DS, HD, ASSD, and standard deviations for all model segmentations are reported in Supplementary Material, Table 1. Box plots showing the evaluation metric distributions are provided in Fig. 5.

In Table 2, DS values are reported per model per cross-section location (AAo, aortic arch [AArch], and descending aorta [DAo]). All seven models reached good DS on all locations, and model 1 achieved the best results (DS > 0.9). DS and ICC values of net flow and peak velocity per location are listed in Tables 2 and 3, Supplementary Material.

**Table 2:** Dice score (mean ± standard deviation) for every model per aortic segment. Best DS values per location and set are in bold

	Dice score	Model 1 (all)	Model 2 (healthy)	Model 3 (BAV)	Model 4 (vendor 1)	Model 5 (male)	Model 6 (age 20-60)	Model 7 (3T)
Test	AAo	0.901 ± 0.041	0.901 ± 0.037	0.907 ± 0.038	**0.913 ± 0.028**	0.911 ± 0.029	0.895 ± 0.052	0.911 ± 0.039
	AArch	0.902 ± 0.040	0.908 ± 0.031	0.896 ± 0.044	0.911 ± 0.035	**0.912 ± 0.028**	0.901 ± 0.037	0.902 ± 0.046
	DAo	0.901 ± 0.044	0.910 ± 0.027	0.902 ± 0.031	0.904 ± 0.038	**0.910 ± 0.027**	0.900 ± 0.040	0.901 ± 0.052
Unrepresented	AAo		0.839 ± 0.084	0.873 ± 0.095	0.872 ± 0.087	0.893 ± 0.061	**0.893 ± 0.054**	0.839 ± 0.120
	AArch		0.850 ± 0.090	0.888 ± 0.039	0.87 ± 0.072	**0.894 ± 0.046**	0.891 ± 0.054	0.853 ± 0.097
	DAo		0.861 ± 0.091	**0.894 ± 0.038**	0.872 ± 0.073	0.892 ± 0.044	0.893 ± 0.052	0.855 ± 0.092
Evaluation	AAo	**0.914 ± 0.038**	0.844 ± 0.104	0.896 ± 0.065	0.891 ± 0.068	0.901 ± 0.057	0.889 ± 0.062	0.876 ± 0.080
	AArch	**0.909 ± 0.042**	0.838 ± 0.114	0.907 ± 0.041	0.877 ± 0.076	0.903 ± 0.043	0.892 ± 0.062	0.870 ± 0.079
	DAo	**0.911 ± 0.034**	0.855 ± 0.094	0.906 ± 0.037	0.891 ± 0.052	0.902 ± 0.038	0.888 ± 0.057	0.870 ± 0.086

*AAo* ascending aorta*, AArch* aortic arch*, DAo* descending aorta*, BAV* bicuspid aortic valve

**Fig. 6:**
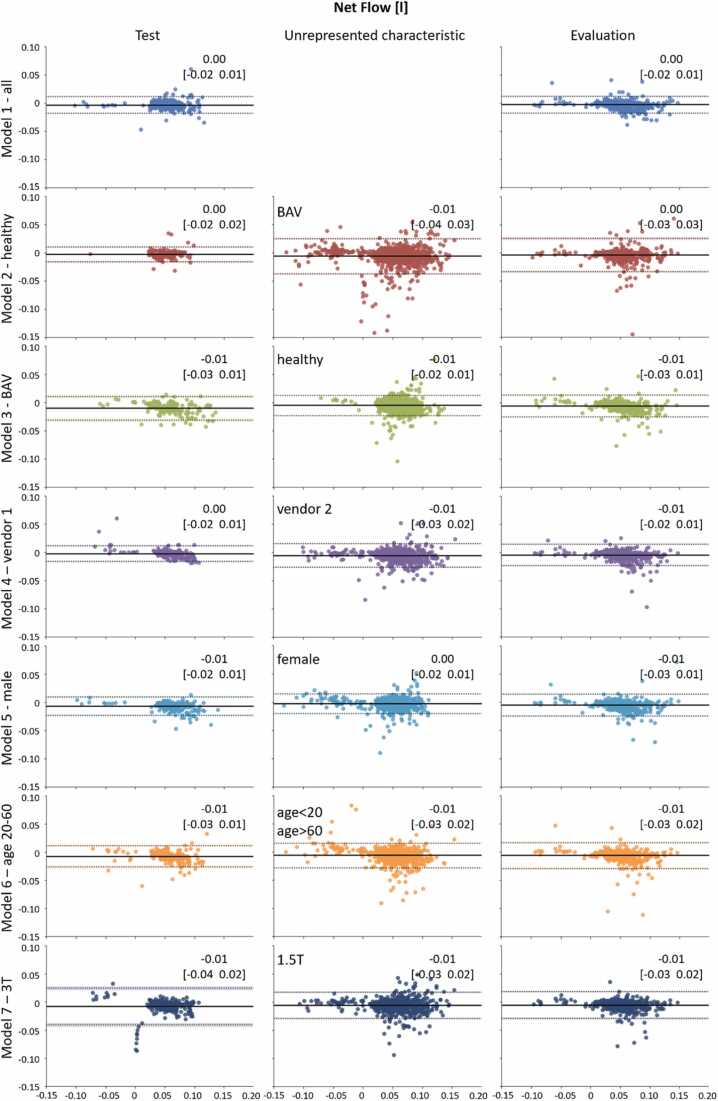
Bland-Altman plots showing automatic-manual segmentations’ agreement of net flow for models 1 to 7. Estimated biases (mean difference) and 95% limits of agreement (average difference ± 1.96 SD of the difference) are shown by continuous and dotted lines, and the values are reported in the right-upper corner of each plot. Biases and limits of agreements are reported in the Supplementary Material. The x and y axes represent mean and difference (CNN − manual) of the net flow in liters resulting from manual and CNN segmentation, respectively. *CNN* convolutional neural network

Flow (net flow, forward flow, and backward flow) and maximum velocity curves were computed for all model segmentations. Bland-Altman plots for net flow on the model’s specific test and unrepresented characteristic sets as well as on the overall evaluation data are shown in Fig. 6. The Bland-Altman analysis for maximum velocity is reported in the Supplementary Material, Fig.2. For both, net flow (through-plane flow) and peak velocity, no clinically relevant bias was found. All seven segmentation models showed good agreement with the expert annotations on their test sets. Net flow and velocity results showed more random fluctuation in the values for the unrepresented datasets. The underrepresentation of healthy subjects, female subjects, and young or very old subjects had less influence on flow parameter agreement of the resulting models than the neglection of vendors or field strengths. Flow results were strongly correlated than the peak velocity, which resulted in more disperse data. On the overall evaluation set, which contained pathologic and postoperative data (POSTOP), the best agreement between the automatic segmentation and the manual ground truth net flow was found for model 1 (bias [limits of agreements (LoA)] = -0.003 [−0.018, 0.012] L). All models achieved comparable results for net flow. For peak velocity, the lowest bias (-0.002 m/s) was achieved by model 4 (vendor 1) followed by model 7 (3T).

Bland-Altman analysis was performed on a subset of the model 1 test dataset to compare intra- and inter-observer variability for net flow (Bias [LoA]: 0.0037 [−0.033, 0.041] L and 0.0042 [−0.032, 0.041] L for intra- and inter-observer, respectively), and for peak velocity (Bias [LoA]: 0.017 [−0.243, 0.277] m/s and −0.009 [−0.229, 0.211] m/s for intra- and inter-observer, respectively).

Additionally, the ICC values and confidence intervals are reported in Table 3. According to the definition by Koo et al. [39] (ICC <0.5: poor, 0.5–0.75: moderate, 0.75–0.9: good, and ICC >0.9 excellent correlation), we found most of the correlation to be excellent except:

• Flow on the test dataset of model 7 (3T) showed a good correlation.
• On their respective unrepresented datasets, model 2 (healthy), 4 (vendor 1), and 7 (3T) showed good correlation for the velocity, and for model 3 (BAV) flow correlation was reported good.
• On the evaluation dataset, velocity correlation was found good for model 2 (healthy), 4 (vendor 1), and 7 (3T), and flow correlation was good for model 2 (healthy).

**Table 3:** ICC values and confidence intervals for net flow and peak velocity for models 1 to 7 on their test and unrepresented characteristic sets (unrepr) as well as the overall evaluation set.

*ICC* interclass correlation coefficient*, BAV* bicuspid aortic valve

The cells are color-coded following the definition by [39], white for excellent and yellow for good correlation. Best values within models are in bold

The ICC values and confidence intervals were computed also on a subset of model 1 test set for intra- and inter-observer variability analysis for both net flow (ICC [CI]: 0.779 [0.69, 0.84] for both intra- and inter-observer) and peak velocity (ICC [CI]: 0.981 [0.97, 0.99] and 0.973 [0.96, 0.98] for intra- and inter-observer, respectively).

To better understand the generalizability of our models based on different stenotic aortic valve morphology and POSTOP, the metrics and the flow parameters were analyzed specifically for the overall evaluation set (unicuspid or tricuspid stenotic aortic valve and postoperative). In Table 4, the DS values and the ICC for net flow and peak velocity are reported for stenotic TAV, stenotic unicuspid aortic valve (UAV), and POSTOP. Excellent correlation for net flow and the peak velocity was found in all groups. The DS was found acceptable for all the models on all subclasses of the overall evaluation dataset. Worst scores were achieved for TAV patients. The best DS and ICC values were found for model 1. Only the peak velocity ICC was better for model 6 (age).

**Table 4:** Dice score metric and ICC for net flow and peak velocity in the ascending aorta for the overall evaluation dataset split into TAV, UAV, and POSTOP.

	Dice score mean			Net flow ICC			Peak velocity ICC		
	TAV	UAV	POSTOP	TAV	UAV	POSTOP	TAV	UAV	POSTOP
Model 1 (all)	**0.889**	**0.906**	**0.937**	**0.965**	**0.979**	**0.996**	**0.941**	0.979	**0.985**
Model 2 (healthy)	0.764	0.832	0.912	0.836	0.943	0.988	0.933	0.973	0.957
Model 3 (BAV)	0.865	0.874	0.926	0.914	0.934	0.986	0.907	0.942	0.951
Model 4 (vendor 1)	0.840	0.896	0.930	0.933	0.970	0.995	0.935	0.879	0.925
Model 5 (male)	0.868	0.894	0.930	0.925	0.948	0.992	0.915	0.958	0.961
Model 6 (age 20−60)	0.846	0.893	0.923	0.874	0.877	0.992	0.913	**0.989**	0.955
Model 7 (3T)	0.817	0.878	0.923	0.882	0.949	0.992	0.920	0.932	0.950

*ICC* interclass correlation coefficient*, TAV* tricuspid aortic valve*, UAV* unicuspid aortic valve*, POSTOP* postoperative

Best Dice score values and ICC are in bold

Bland-Altman plots for systolic area in mm^2^ on the model’s specific test and unrepresented characteristic sets and the overall evaluation set are reported in the Supplementary Material, Fig. 3. We observed a general underestimation of the systolic area, and model 1 achieved the lowest bias (−65.1 mm^2^) on the evaluation test.

**Fig. 7:**
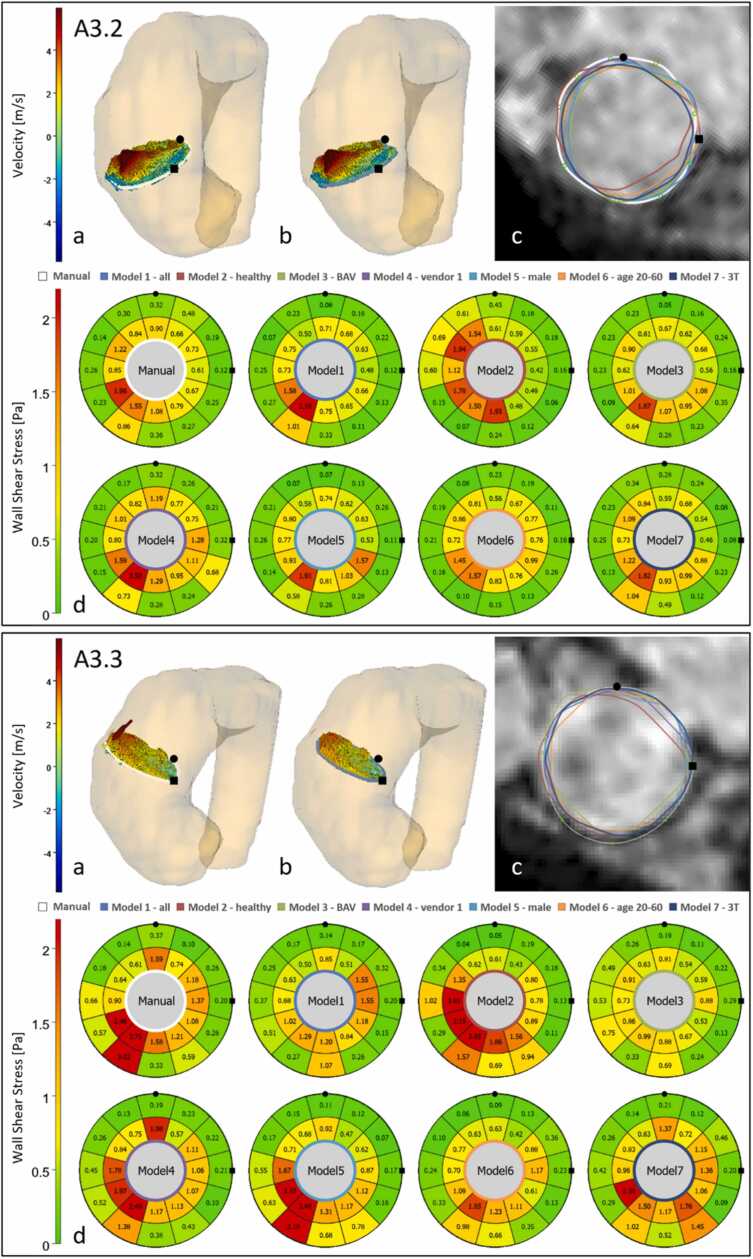
Example of systolic segmentation results on two cross-sections A3.2 (upper) and A3.3 (bottom) of a tricuspid stenotic valve patient from site 5 in the evaluation set. (78-year old male patient, scanned with Philips Achieva 1.5T, venc = 600 cm/s). The 3D visualizations show systolic velocity vectors for manual segmentation (a) and for model 1 automatic segmentation (b) and the segmentation results for every model on the magnitude image in systole (c). The bull’s-eye plots (BEP) for axial WSS computed for the manual and automatic contours (d). Note that BEPs depict two WSS metrics: maximum axial WSS (inner BEP values) and mean WSS in systole (outer BEP values). The circle and square glyphs indicate the orientation. *Venc* velocity encoded, *3D* three-dimensional, *WSS* wall shear stress

Fig. 7 displays the systolic segmentation results of the seven models on two cross-sections of the AAo (A3.2 and A3.3) of an evaluation dataset of a tricuspid stenotic aortic valve patient. The segmentation results of model 2 (trained only on healthy subjects) in both cross-sections differ most from the ground truth. The bulls-eye-plots illustrate the effect of the vessel segmentation differences on the axial WSS. In cross-section A3.2, high velocities occur in the lumen center. Axial WSS values of the model 1 segmentation are higher than those of the manual contour, which is located further away from the lumen center. Conversely, in cross-section A3.3, high velocities are observed close to the lumen border, resulting in strong differences for the WSS values in the corresponding segments for the different segmentations.

## 4 Discussion

The most important result of the presented work is the analysis of the impact of training data characteristics representations on the application performance of a state-of-the-art segmentation model on heterogeneous 4D flow CMR data of the aorta. Our analysis showed a stronger influence of scan-related characteristics (different vendors and field strength) than of patient characteristics, such as age and gender, on the segmentation performance. This is illustrated in Fig. 5 (Table 1, Supplementary Material), showing e.g. that the models trained on male subjects only or on subjects aged 20–60 perform well also for datasets of female subjects (DS = 0.89, HD = 2.88, and ASSD = 0.72) and younger or older subjects (DS = 0.89, HD = 3.05, and ASSD = 0.78).

While model 2, which was trained only on data from healthy subjects, performed worst on both unrepresented characteristics (BAV) as well as on the evaluation set, model 3, which was trained on BAV datasets, also performed well on healthy subjects’ data (unrepresented characteristic dataset in Fig. 5 and values in Table 1, Supplementary Material). This could be explained by the presence of cross-sections of the DAo with relatively laminar flow in the training set of model 3. This was in line with the slightly lower DS of the model in the AAo in both the unrepresented and overall evaluation sets (Table 2). The performance difference between these two models (healthy and BAV trained) could also be influenced by the inclusion of a wider variety of velocity encoding settings in the BAV datasets, resulting in an improvement in the generalization of the model. Furthermore, the valve pathologies represented in the overall evaluation set can cause flow profiles that are similar to those of bicuspid valves and therefore model 3 reached the second-best DS there. On the overall evaluation set, model 1, which was trained on all the data, achieved the best DS (0.911 ± 0.039), HD (2.797 ± 1.166 mm), and ASSD (0.655 ± 0.267 mm). No substantial differences in segmentation metrics were found between cross-sectional planes, with model 1 (all) achieving the best DS values (>0.909) for all locations.

The Bland-Altman analysis reported good agreement for all models on all datasets for net flow, with worse results on the unrepresented characteristics dataset. We considered absolute values of net flow bias <0.004 L and peak velocity bias <0.069 m/s clinically not relevant since these values are smaller than the ones observed in scan-rescan analysis in [9] and [30], respectively. No clinically relevant bias was found for net flow for model 1 (Supplementary Material); the lowest values were found for model 1 on the overall evaluation set (−0.003 [−0.018, 0.012], Table 4, Supplementary Material). Peak velocity Bland-Altman analysis showed a weaker agreement, obtaining clinically relevant biases for model 2 (healthy) on the unrepresented characteristic set and for model 3 (BAV) and model 5 (male) on the overall evaluation set. On their corresponding unrepresented characteristic sets, model 5 (male) and 6 (age) achieved excellent ICC values, showing the positive effect of the good segmentation agreement on the clinical parameter reproducibility. On the overall evaluation set, model 1 (all), 3 (BAV), 5 (male) and model 6 (age) achieved excellent ICCs for both net flow and peak velocity. No clinically relevant bias was found for model 1 (all) in neither the net flow nor the peak velocity, and excellent correlation found for both parameters, indicating that its segmentation might be more robust with regard to the inclusion of lumen with low flow velocities than the other models, which had a negative bias.

There were no relevant differences between segmentation results provided for datasets of patients with different stenotic aortic valve morphologies and postoperative data by the differently trained seven models, only a slightly lower DS for TAV (Table 4).

Model 1 trained on the complete heterogeneous dataset showed good performance for the different manufacturers, pathologies, and cross-section positions for all age groups, achieving an excellent DS of 0.911 ± 0.039. Model 1 performance is comparable to the one obtained by the multi-site 3D DL approach proposed by Fujiwara et al. [26], for both DS (0.911 vs 0.915) and net flow bias and LoA in liters (−0.003 [−0.018, 0.012] vs 0.002 [−0.011, 0.015]). Model 1 automatic segmentation (bias [LoA]: −0.004 [−0.018, 0.011] and ICC 0.954) provided comparable net flow bias results and better ICC compared to the intra-observer analysis (bias [LoA]: 0.0036 [−0.033, 0.041] and ICC 0.778) and the inter-observer analysis (bias [LoA]: 0.0042 [−0.032, 0.041] and ICC 0.778). The automatic segmentation provided flow and velocity curves comparable with expert analyses, showing ICC values comparable to those observed in literature for inter-user agreement [16]. This strengthens the hypothesis that the performance of U-net-based segmentation models depends on the similarity of the dataset to be processed and the training set, thus cross-sectional 4D flow CMR segmentation models can be applied to new patient groups if their characteristics do not result in completely new cross-sectional velocity and magnitude intensity patterns.

The negative bias of the lumen area in systole corresponds to the negative bias observed for the net flow. In the experiments by Zimmermann et al. [13] contour shrinking resulted in an increase in WSS. The examples in Fig. 7 illustrate that the negative area bias does not necessarily mean that the segmentations with a smaller area are inside the expert lumen segmentations. Furthermore, we observe flow patterns, for which the velocities can decrease toward the lumen center. We therefore assume that the WSS values determined with the segmentations by the best model are not necessarily correct or adjustable by a lumen expansion.

## 5 Limitations

The most important limitation of this work is the difference in the number of subjects provided by the different sites. The evaluation was performed using data from the site which was least represented in the main dataset. Although different aortic valve morphologies were analyzed, only stenotic patients were included in the main and evaluation datasets. The impact of including additional pathologies remains to be investigated. In addition, just one exemplary popular DL architecture was analyzed.

## 6 Conclusion

We investigated the dependency of a state-of-the-art segmentation model’s performance for aortic cross-section segmentation in 4D flow CMR on the representation of patient characteristics as well as scanner and imaging sequence properties in the training data. We found that the field strength and aortic valve pathologies were the most important characteristics for training a widely applicable segmentation model for reproducible flow quantification in the aorta. This study underlines the importance of model cards reporting the properties of the training data of machine learning models, so that the users can assess the suitability of the models for processing their datasets. In the future, we intend to address time-resolved 3D segmentation methods, enabling further analysis such as pressure maps and WSS, and extending our investigation beyond the constraints of 2D+t segmentation.

## Funding

This work was partially funded by the 10.13039/501100001659German Research Foundation (GRK2260, BIOQIC). S.K. received support from the DZHK (German Center for Cardiovascular Research), Partner Site Berlin. S.K. was supported by an unrestricted research grant from Philips Healthcare. L.W., T.K., S.K., and A.He. were partially funded by the 10.13039/501100001659Deutsche Forschungsgemeinschaft (DFG, 10.13039/501100001659German Research Foundation) - SFB-1470 - B06 and SFB 1340 - A01. A.Ha. was supported by the Berta-Ottenstein-Program for Advanced Clinician Scientists, Faculty of Medicine, 10.13039/501100002714University of Freiburg, Germany.

## Author contributions

C.Ma., M.H., L.W., A.He.: conceptualization. S.N., L.J., H.S., C.Me., A.Ha., J.E., S.K., P.B., R.F.T., J.S.M.: data collection and curation. C.Ma., M.H., A.He.: data processing. All authors read and approved the final manuscript.

## Ethics approval and consent

The studies involving human participants were reviewed and approved by the local ethics board (Albert-Ludwigs-Universität Freiburg, Charité - Universitätsmedizin Berlin, Technical University of Munich, University Medical Center Hamburg-Eppendorf). Written informed consent was obtained from all patients/participants.

## Declaration of competing interests

Chiara Manini reports financial support was provided by German Research Foundation. Sebastian Kelle reports financial support was provided by German Center for Cardiovascular Disease. Anja Hennemuth reports financial support was provided by German Research Foundation. Titus Kuehne reports financial support was provided by German Research Foundation. Sebastian Kelle reports financial support was provided by German Research Foundation. Andreas Harloff reports financial support was provided by University of Freiburg Faculty of Medicine. Sebastian Kelle reports a relationship with Philips Healthcare that includes funding grants. The other authors declare that they have no known competing financial interests or personal relationships that could have appeared to influence the work reported in this paper.

## Data Availability

The datasets generated and analyzed during the current study are not publicly available but are available from the corresponding author on reasonable request. The general trained segmentation model is publicly available under CC-BY-NC-SA.
