# Supplementary material for: Impact of training data composition on the generalizability of convolutional neural network aortic cross-section segmentation in four-dimensional magnetic resonance flow imaging
Source: J Cardiovasc Magn Reson. 2024 Aug 8;26(2):101081. doi: 10.1016/j.jocmr.2024.101081 (PMC11422555; doi:10.1016/j.jocmr.2024.101081)
Supplement: Supplementary file 1 — Supplementary material [file mmc1.pdf]

Impact of training data composition on the generalizability of convolutional neural network aortic cross-section segmentation in 4D Flow MRI

Chiara Manini [a, b,*], Markus Hüllebrand [a, b, c], Lars Walczak [a, b, c], Sarah Nordmeyer [d], Lina Jarmatz [a], Titus Kuehne [a, b, e], Heiko Stern [f], Christian Meierhofer [f], Andreas Harloff [g], Jennifer Erley [e, h], Sebastian Kelle [e, h], Peter Bannas [i], Ralf Felix Trauzeddel [e, j, k, l], Jeanette Schulz-Menger [e, j, k, m], Anja Hennemuth [a, b, c, e, i]

a. Deutsches Herzzentrum der Charité (DHZC), Institute of Computer-assisted Cardiovascular Medicine, Berlin, Germany
b. Charité – Universitätsmedizin Berlin, corporate member of Freie Universität Berlin and Humboldt Universität zu Berlin, Berlin, Germany
c. Fraunhofer MEVIS, Berlin, Germany
d. Department of Diagnostic and Interventional Radiology, Tübingen University Hospital, Tübingen, Germany
e. German Center for Cardiovascular Research (DZHK), Partner Site Berlin, Germany
f. Congenital Heart Disease and Pediatric Cardiology, German Heart Center Munich, Germany
g. Department of Neurology and Neurophysiology, University Medical Center Freiburg - Faculty of Medicine, University of Freiburg, Freiburg, Germany
h. Department of Cardiology, Angiology and Intensive Care Medicine, Deutsches Herzzentrum der Charité - Universitätsmedizin Berlin, Augustenburger Platz 1, 13353 Berlin, Corporate Member of Freie Universität Berlin and Humboldt - Universität zu Berlin, Berlin, Germany
i. Department of Diagnostic and Interventional Radiology and Nuclear Medicine, University Medical Center Hamburg-Eppendorf, Germany
j. Charité – Universitätsmedizin Berlin, corporate member of Freie Universität Berlin and Humboldt-Universität zu Berlin, ECRC Experimental and Clinical Research Center, Lindenberger Weg 80, 13125 Berlin, Germany
k. Working Group on Cardiovascular Magnetic Resonance, Experimental and Clinical Research Center, a joint cooperation between the Charité – Universitätsmedizin Berlin and the Max-Delbrück-Center for Molecular Medicine, Berlin, Germany
l. Charité – Universitätsmedizin Berlin, corporate member of Freie Universität Berlin and Humboldt-Universität zu Berlin, Department of Anesthesiology and Intensive Care Medicine, Charité Campus Benjamin Franklin, Hindenburgdamm 30, 12203 Berlin, Germany
m. Department of Cardiology and Nephrology, Helios Hospital Berlin-Buch, Berlin, Germany

*. Corresponding author: Chiara Manini, chiara.manini@dhzc-charite.de

Supplementary material

Model evaluation and Statistical analysis

- Dice Score (DS): Spatial overlap between regions (X and Y).

$$DS(X,Y) = \frac{2 \times |X \cap Y|}{|X| + |Y|}$$

- Hausdorff distance (HD): Maximum distance from a point in one set to the closest point in the other set. Calculated as:

$$HD(X,Y) = \max(h(X,Y), h(Y,X))$$

where:

$$h(A,B) = \max_{a \in A}\left(\min_{b \in B}(d(a,b))\right)$$

- Average symmetric surface distance (ASSD): Average of the closest distances from all the surface points to the other surface, and vice versa. Computed as follows:

$$ASSD(X,Y) = \frac{1}{2}\left(\frac{\sum_{x=1}^{X} \min_{y \in Y}(\|x-y\|)}{|X|} + \frac{\sum_{y=1}^{Y} \min_{x \in X}(\|x-y\|)}{|Y|}\right)$$

Flow and velocity calculation

Flow I(t) and velocity v(t) curve are computed as:

$$v(x) = \frac{1}{|A(t)|}\int_{A(t)} \|v(x,y,t)\| \, dA$$

$$I(t) = \int_{A(t)} \langle v(x,y,t), n \rangle \, dA$$

With:

- $\|v\|$ the magnitude of the velocity vector v
- $v(x,y,t)$ the velocity vector in the point (x,y) at time t

- $|A(t)|$ the area of the segmentation on timeframe t
- n normal vector of the cross-sectional plane
- And $\langle a, b \rangle$ denotes the scalar product between the vectors a and b.

Metrics values

Table 1. ICC values and confidence intervals for minimum and maximum diameters over time for model 1-7 on their test and unrepresented datasets as well as the evaluation dataset. The cells are color coded following the definition by Koo et al. , white for excellent, yellow for good and orange for moderate correlation.

	ICC2	Model 1 (all)	Model 2 (healthy)	Model 3 (BAV)	Model 4 (vendor 1)	Model 5 (male)	Model 6 (age 20-60)	Model 7 (3T)
Test	Diameter min	0.854 [0.73 0.91]	0.817 [0.75 0.86]	0.822 [0.10 0.94]	**0.869** [0.75 0.92]	0.854 [0.57 0.93]	0.802 [0.24 0.92]	0.825 [0.53 0.91]
	Diameter max	0.806 [0.73 0.86]	0.752 [0.68 0.81]	0.817 [0.31 0.93]	**0.864** [0.80 0.90]	0.785 [0.66 0.86]	0.792 [0.25 0.92]	0.834 [0.64 0.91]
Unrepr	Diameter min		0.800 [0.59 0.89]	0.691 [0.63 0.74]	**0.843** [0.72 0.90]	0.817 [0.76 0.85]	0.787 [0.28 0.91]	0.783 [0.62 0.86]
	Diameter max		0.823 [0.74 0.87]	0.574 [0.54 0.60]	**0.823** [0.70 0.89]	0.752 [0.72 0.78]	0.782 [0.38 0.90]	0.726 [0.61 0.80]
Evaluation	Diameter min	**0.842** [0.44 0.93]	0.733 [0.38 0.86]	0.765 [0.28 0.90]	0.799 [0.55 0.89]	0.765 [0.24 0.90]	0.745 [0.80 0.90]	0.679 [0.37 0.82]
	Diameter max	**0.823** [0.58 0.91]	0.774 [0.56 0.87]	0.750 [0.47 0.86]	0.802 [0.61 0.88]	0.715 [0.39 0.84]	0.743 [0.09 0.90]	0.657 [0.44 0.78]

We define successful segmentations as those with DS>0.8 to illustrate the success rate in dependence of plane location (Figure 1). Success rates are similar within the different locations for all the models, and model 1 exhibits excellent success rate in all the locations.

DS > 0.8 [%]	Model 1 (all)	Model 2 (healthy)	Model 3 (BAV)	Model 4 (vendor 1)	Model 5 (male)	Model 6 (age 20-60)	Model 7 (3T)
Unrepresented	-	78	96	86	95	93	80
Separated Evaluation	98	74	97	88	97	92	82

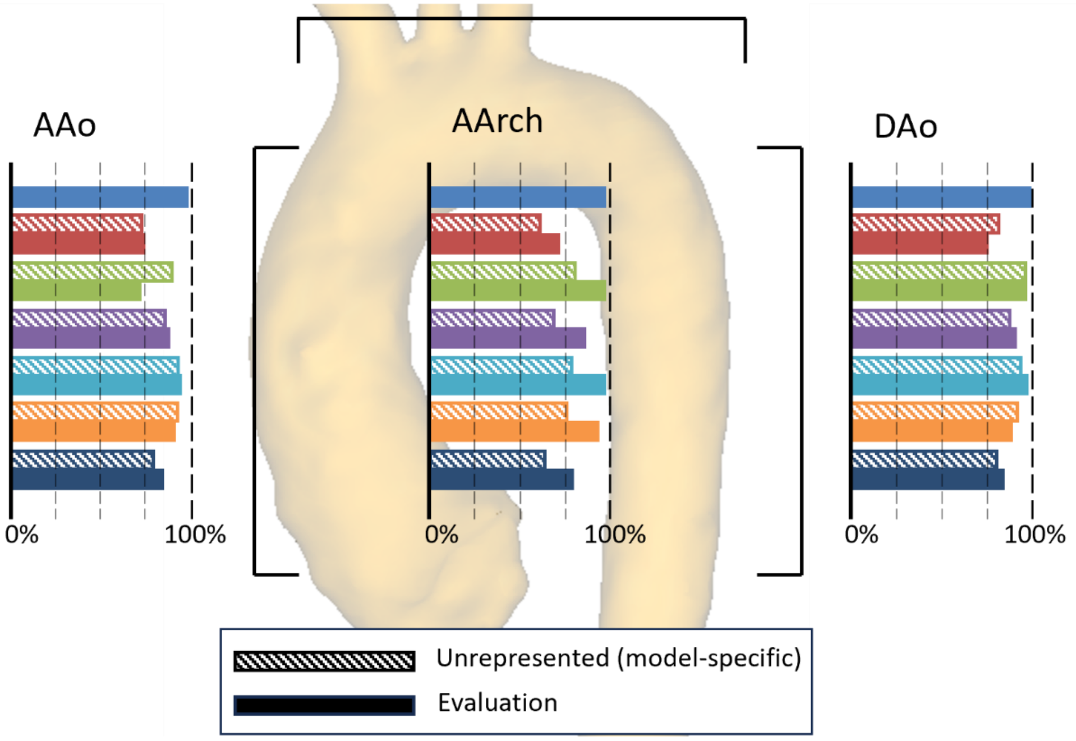

Figure 1. Percentage of successful cross-sectional vessel segmentation (DS>0.8). In the table on the top the percentage of successful segmentation planes over the full corresponding dataset are reported (all locations). The color bars represent the percentage of the successfully segmented planes in the locations AAo: ascending aorta, AArch: aortic arch, and DAo: descending aorta.

Table 2. Mean Dice Score on the overall evaluation set for every model grouped by cross section location. The best dice score per location is in bold.

DS	Model1	Model2	Model3	Model4	Model5	Model6	Model7
A3.1	**0.900**	0.839	0.869	0.872	0.883	0.866	0.853
A3.2	**0.918**	0.817	0.899	0.895	0.897	0.893	0.874
A3.3	**0.925**	0.877	0.919	0.907	0.924	0.909	0.901
B1	**0.921**	0.873	0.918	0.896	0.914	0.910	0.899
B2	**0.904**	0.848	0.900	0.879	0.901	0.903	0.887
B3	**0.910**	0.847	0.900	0.870	0.902	0.894	0.862
B4.1	**0.894**	0.812	0.897	0.862	0.893	0.880	0.850
B4.2	**0.910**	0.815	0.908	0.869	0.903	0.877	0.854
B4.3	0.912	0.841	**0.913**	0.885	0.905	0.894	0.870
D1.1	**0.920**	0.855	0.915	0.896	0.909	0.894	0.881
D1.2	**0.913**	0.842	0.907	0.888	0.902	0.886	0.860
D1.3	**0.902**	0.867	0.897	0.889	0.895	0.884	0.869

Table 3. Through flow (accumulated over time) and peak velocity (over time) interclass coefficients for every model grouped by cross section location on the overall evaluation set. The cells are color coded following the definition by Koo et al. , white for excellent, yellow for good, orange for moderate correlation and red for poor.

**Net Flow ICC**	**Model1**	**Model2**	**Model3**	**Model4**	**Model5**	**Model6**	**Model7**
A3.1	0.981	0.954	0.942	0.982	0.980	0.928	**0.994**
A3.2	**0.956**	0.629	0.881	0.871	0.871	0.792	0.774
A3.3	**0.954**	0.939	0.914	0.941	0.919	0.899	0.867
B1	**0.970**	0.939	0.933	0.956	0.953	0.942	0.932
B2	**0.938**	0.837	0.907	0.888	0.889	0.847	0.888
B3	**0.956**	0.875	0.928	0.925	0.934	0.910	0.928
B4.1	**0.937**	0.776	0.915	0.873	0.920	0.861	0.915
B4.2	**0.949**	0.826	0.923	0.895	0.920	0.912	0.858
B4.3	**0.972**	0.962	0.969	0.957	0.961	0.963	0.947
D1.1	**0.984**	0.958	0.975	0.977	0.975	0.976	0.957
D1.2	**0.948**	0.897	0.946	0.942	0.932	0.917	0.900
D1.3	0.951	0.940	0.919	**0.960**	0.930	0.924	0.916
**Velocity ICC**	**Model1**	**Model2**	**Model3**	**Model4**	**Model5**	**Model6**	**Model7**
A3.1	0.994	0.992	0.995	0.997	**0.998**	0.994	0.995
A3.2	**0.922**	0.873	0.860	0.902	0.882	0.893	0.843
A3.3	0.831	**0.841**	0.727	0.819	0.731	0.737	0.809
B1	**0.966**	0.926	0.951	**0.966**	0.954	0.954	0.886
B2	**0.942**	0.842	0.935	0.865	0.934	0.930	0.904
B3	0.844	0.858	0.843	**0.899**	0.837	0.833	0.768
B4.1	0.701	0.408	0.857	0.706	0.840	**0.905**	0.626
B4.2	**0.859**	0.756	0.779	0.664	0.765	0.812	0.799
B4.3	0.702	**0.714**	0.699	0.526	0.702	0.694	0.678
D1.1	0.804	0.821	0.976	0.920	**0.978**	0.977	0.680
D1.2	0.572	0.218	0.641	0.645	**0.694**	0.392	0.356
D1.3	0.813	0.352	0.803	**0.858**	0.797	0.575	0.733

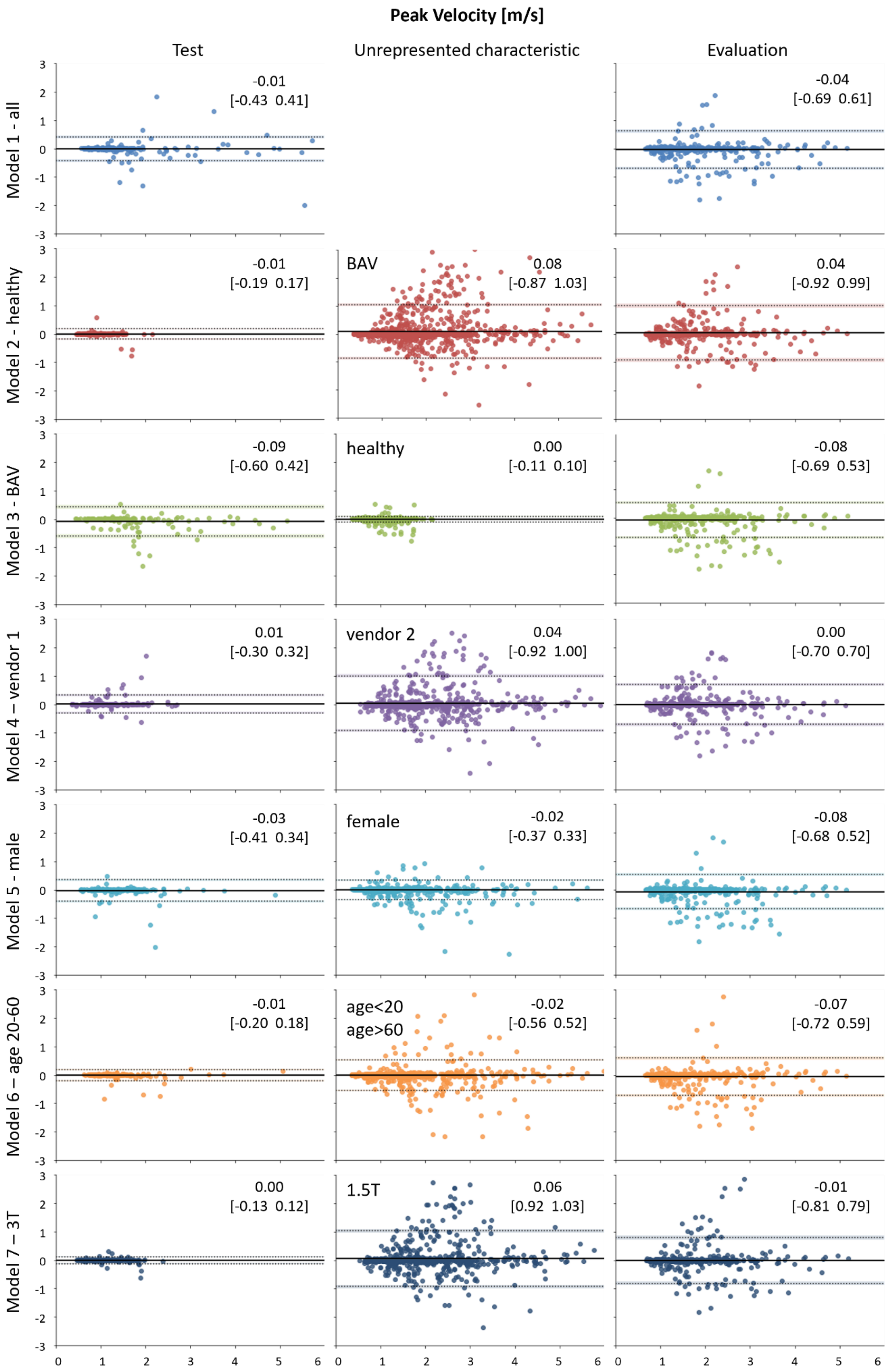

Figure 2. Bland-Altman plots showing automatic-manual segmentations agreement of peak velocity for models 1 to 7. Estimated biases (mean difference) and 95% limits of agreement (average difference ± 1.96 SD of the difference) are shown by continuous and dotted lines and the values are reported in the right-upper corner of each plot. Biases and limits of agreements are reported in the supplementary material. X and y axis represent mean and difference (CNN – manual) of the peak velocity in m/s resulting from manual and CNN segmentation, respectively.

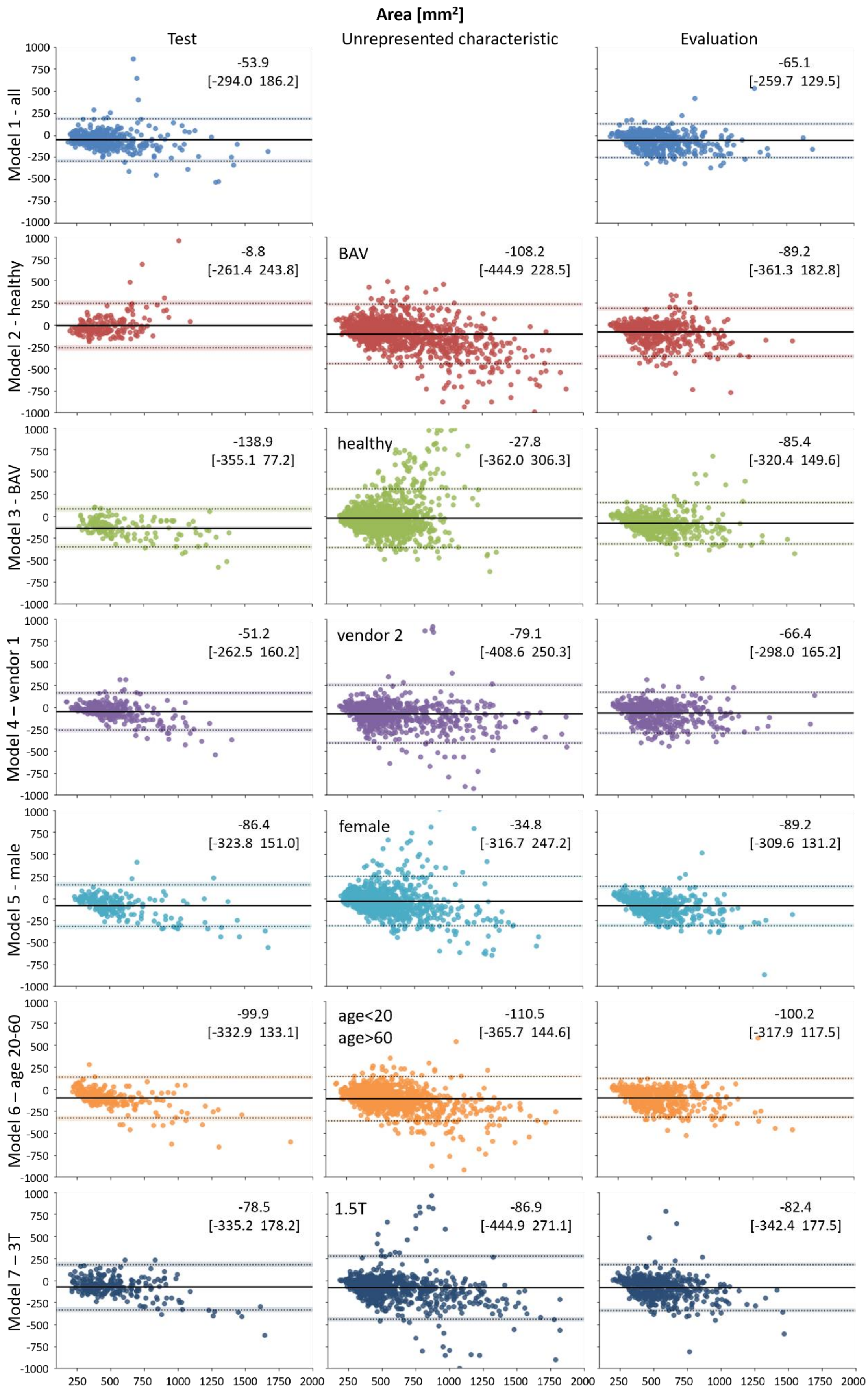

Figure 3. Bland-Altman plots showing automatic-manual segmentations agreement of systolic area in mm² for models 1 to 7. Estimated biases (mean difference) and 95% limits of agreement (average difference ± 1.96 SD of the difference) are shown by continuous and dotted lines and the values are reported in the right-upper corner of each plot. Biases and limits of agreements are reported in the supplementary material. X and y axis represent mean and difference (CNN – manual) of the peak velocity in mm² resulting from manual and CNN segmentation, respectively.

Table 4. Overview of all the parameters computed for each model on the 3 datasets (test, unrepresented characteristic, and overall evaluation set). In the table are reported: mean ± standard deviation for dice score, hausdorff distance (HD) and asymmetric surface distance (ASSD); bias [limits of agreements (LoA)] and interclass coefficient (ICC) [confidence intervals (CI)] for throughflow in liters and peak velocity in m/s. The best values across the different models are in bold.

					**Through Flow**				**Peak velocity**				**Systolic area**			
		**Dice Score**	**HD [mm]**	**ASSD [mm]**	**Bias [l]**	**LoA [l]**	**ICC**	**CI**	**Bias [m/s]**	**LoA [m/s]**	**ICC**	**CI**	**Bias [mm^2]**	**LoA [mm^2]**	**ICC**	**CI**
**TEST**	**Model 1 (all)**	0.902 ± 0.042	2.692 ± 1.015	0.674 ± 0.276	-0.004	[-0.018 0.011]	0.954	[0.92 0.97]	-0.012	[-0.430 0.405]	0.963	[0.95 0.97]	-53.91	[-294.0 186.2]	0.848	[0.76 0.90]
	**Model 2 (healthy)**	0.906 ± 0.032	**2.493 ± 0.696**	**0.610 ± 0.210**	**-0.003**	[-0.016 0.010]	0.923	[0.86 0.95]	-0.007	[-0.185 0.171]	0.952	[0.94 0.96]	**-8.82**	[-261.4 243.8]	0.756	[0.69 0.81]
	**Model 3 (BAV)**	0.901 ± 0.040	3.132 ± 1.137	0.730 ± 0.270	-0.011	[-0.031 0.010]	0.919	[0.56 0.97]	-0.088	[-0.595 0.419]	0.954	[0.93 0.97]	-138.9	[-355.1 77.2]	0.821	[0.11 0.94]
	**Model 4 (vendor 1)**	0.909 ± 0.034	2.573 ± 0.850	0.631 ± 0.230	-0.003	[-0.017 0.011]	**0.965**	[0.95 0.98]	0.014	[-0.296 0.324]	0.932	[0.91 0.95]	-51.16	[-262.5 160.2]	0.856	[0.76 0.91]
	**Model 5 (male)**	**0.911 ± 0.028**	2.664 ± 0.928	0.644 ± 0.215	-0.008	[-0.024 0.009]	0.946	[0.73 0.98]	-0.034	[-0.412 0.344]	0.938	[0.92 0.95]	-86.36	[-323.8 151.0]	**0.857**	[0.62 0.93]
	**Model 6 (age 20-60)**	0.899 ± 0.042	2.747 ± 1.222	0.681 ± 0.286	-0.008	[-0.027 0.011]	0.915	[0.70 0.96]	-0.014	[-0.204 0.176]	**0.982**	[0.98 0.99]	-99.93	[-332.9 133.1]	0.826	[0.45 0.92]
	**Model 7 (3T)**	0.904 ± 0.046	2.645 ± 0.888	0.658 ± 0.272	-0.009	[-0.041 0.024]	0.866	[0.75 0.92]	**-0.004**	[-0.128 0.120]	0.980	[0.97 0.98]	-78.51	[-335.2 178.2]	0.843	[0.67 0.91]

					**Through Flow**				**Peak velocity**				**Systolic area**			
		**Dice Score**	**HD [mm]**	**ASSD [mm]**	**Bias [l]**	**LoA [l]**	**ICC**	**CI**	**Bias [m/s]**	**LoA [m/s]**	**ICC**	**CI**	**Bias [mm^2]**	**LoA [mm^2]**	**ICC**	**CI**
**UNREPRESENTED**	**Model 2 (healthy)**	0.850 ± 0.089	4.153 ± 2.438	1.107 ± 0.696	-0.006	[-0.037 0.025]	0.917	[0.88 0.94]	0.081	[-0.868 1.031]	0.860	[0.84 0.88]	-108.2	[-444.9 228.5]	0.810	[0.60 0.89]
	**Model 3 (BAV)**	0.886 ± 0.059	3.163 ± 2.102	0.774 ± 0.476	-0.005	[-0.023 0.013]	0.851	[0.70 0.91]	**-0.004**	[-0.106 0.098]	**0.979**	[0.98 0.98]	**-27.81**	[-362 306.3]	0.608	[0.57 0.64]
	**Model 4 (vendor 1)**	0.871 ± 0.076	3.622 ± 1.976	0.931 ± 0.561	-0.006	[-0.026 0.015]	**0.939**	[0.88 0.96]	0.042	[-0.915 0.999]	0.881	[0.86 0.90]	-79.15	[-408.6 250.3]	**0.827**	[0.72 0.88]
	**Model 5 (male)**	**0.893 ± 0.050**	**2.881 ± 1.335**	**0.727 ± 0.348**	**-0.003**	[-0.021 0.014]	0.937	[0.91 0.95]	-0.020	[-0.366 0.327]	0.964	[0.96 0.97]	-34.76	[-316.7 247.2]	0.794	[0.76 0.82]
	**Model 6 (age 20-60)**	0.892 ± 0.053	3.057 ± 1.284	0.778 ± 0.404	-0.007	[-0.028 0.015]	0.907	[0.80 0.95]	-0.018	[-0.557 0.522]	0.951	[0.94 0.96]	-110.5	[-365.7 144.6]	0.800	[0.40 0.91]
	**Model 7 (3T)**	0.850 ± 0.102	4.144 ± 2.905	1.098 ± 0.784	-0.006	[-0.029 0.017]	0.927	[0.86 0.96]	0.056	[-0.918 1.029]	0.879	[0.86 0.89]	-86.94	[-444.9 271.1]	0.785	[0.66 0.85]

		Dice Score	HD [mm]	ASSD [mm]	Through Flow				Peak velocity				Systolic area			
					Bias [l]	LoA [l]	ICC	CI	Bias [m/s]	LoA [m/s]	ICC	CI	Bias [mm^2]	LoA [mm^2]	ICC	CI
EVALUATION	**Model 1 (all)**	**0.911 ± 0.039**	**2.797 ± 1.166**	**0.655 ± 0.267**	**-0.003**	**[-0.018 0.012]**	**0.969**	**[0.95 0.98]**	-0.041	[-0.693 0.611]	0.913	[0.90 0.93]	**-65.10**	[-259.7 129.5]	**0.865**	[0.68 0.93]
	**Model 2 (healthy)**	0.844 ± 0.107	4.002 ± 2.448	1.100 ± 0.761	-0.004	[-0.034 0.025]	0.893	[0.86 0.92]	0.037	[-0.917 0.991]	0.824	[0.79 0.85]	-89.24	[-361.3 182.8]	0.734	[0.48 0.85]
	**Model 3 (BAV)**	0.904 ± 0.048	2.972 ± 1.559	0.702 ± 0.362	-0.006	[-0.025 0.013]	0.938	[0.85 0.97]	-0.079	[-0.688 0.530]	0.917	[0.89 0.93]	-85.36	[-320.4 149.6]	0.796	[0.51 0.89]
	**Model 4 (vendor 1)**	0.884 ± 0.068	3.288 ± 1.662	0.829 ± 0.464	-0.005	[-0.024 0.014]	0.946	[0.90 0.97]	**-0.002**	**[-0.704 0.699]**	0.899	[0.88 0.91]	-66.40	[-298 165.2]	0.825	[0.67 0.89]
	**Model 5 (male)**	0.902 ± 0.046	2.943 ± 1.357	0.710 ± 0.344	-0.005	[-0.025 0.014]	0.944	[0.88 0.97]	-0.077	[-0.678 0.524]	**0.919**	**[0.90 0.94]**	-89.21	[-309.6 131.2]	0.791	[0.44 0.90]
	**Model 6 (age 20-60)**	0.891 ± 0.061	3.112 ± 1.564	0.786 ± 0.435	-0.006	[-0.029 0.017]	0.917	[0.84 0.95]	-0.066	[-0.722 0.590]	0.909	[0.89 0.92]	-100.2	[-317.9 117.5]	0.786	[0.33 0.90]
	**Model 7 (3T)**	0.872 ± 0.081	3.497 ± 1.885	0.917 ± 0.571	-0.006	[-0.030 0.018]	0.918	[0.85 0.95]	-0.007	[-0.808 0.794]	0.871	[0.85 0.89]	-82.41	[-342.4 177.5]	0.754	[0.52 0.86]

Model cards

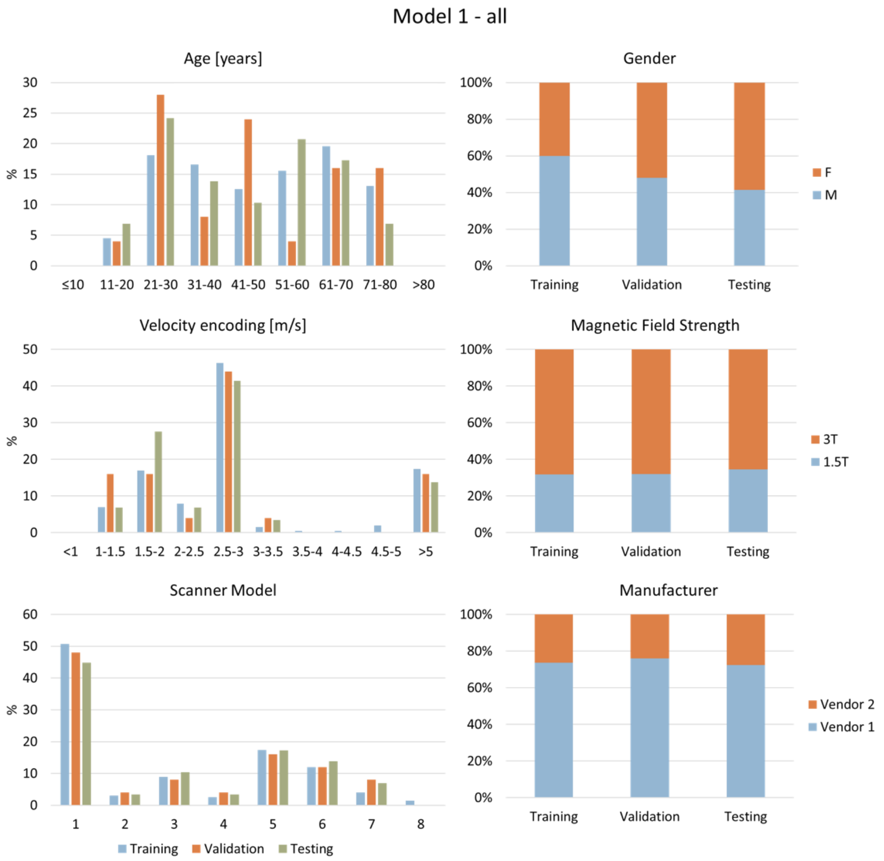

Figure 4. Model 1 (all) card. Detailed statistic information about the model-specific training, validation and testing splits.

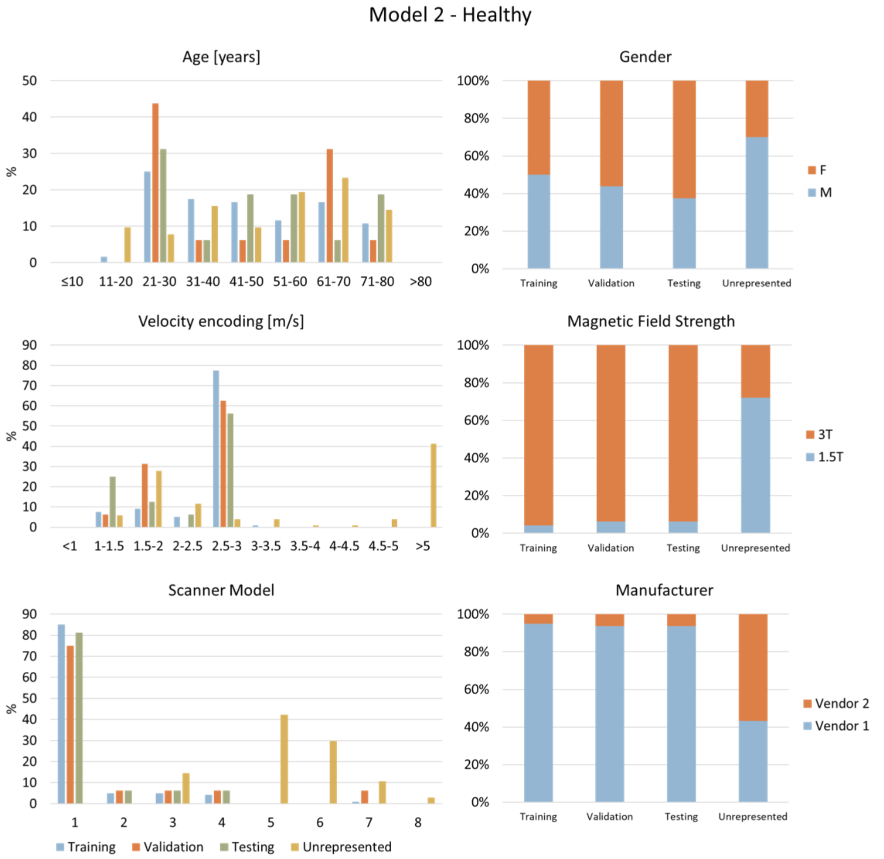

Figure 5. Model 2 (healthy) card. Detailed statistic information about the model-specific training, validation, testing and unrepresented splits.

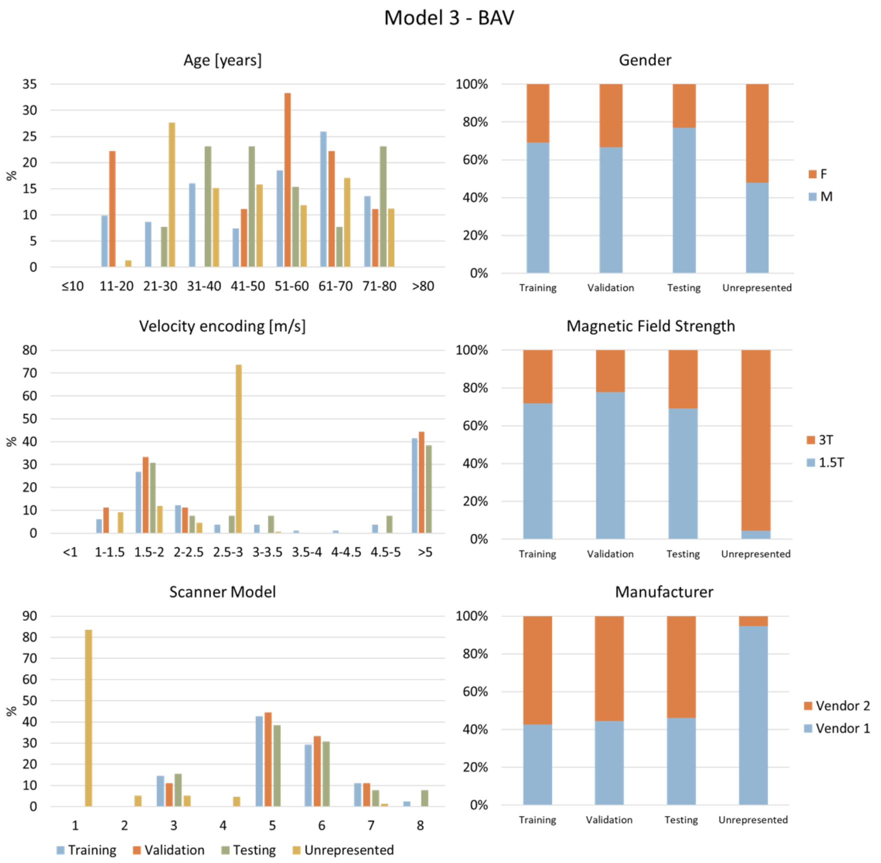

Figure 6. Model 3 (BAV) card. Detailed statistic information about the model-specifictraining, validation, testing and unrepresented splits.

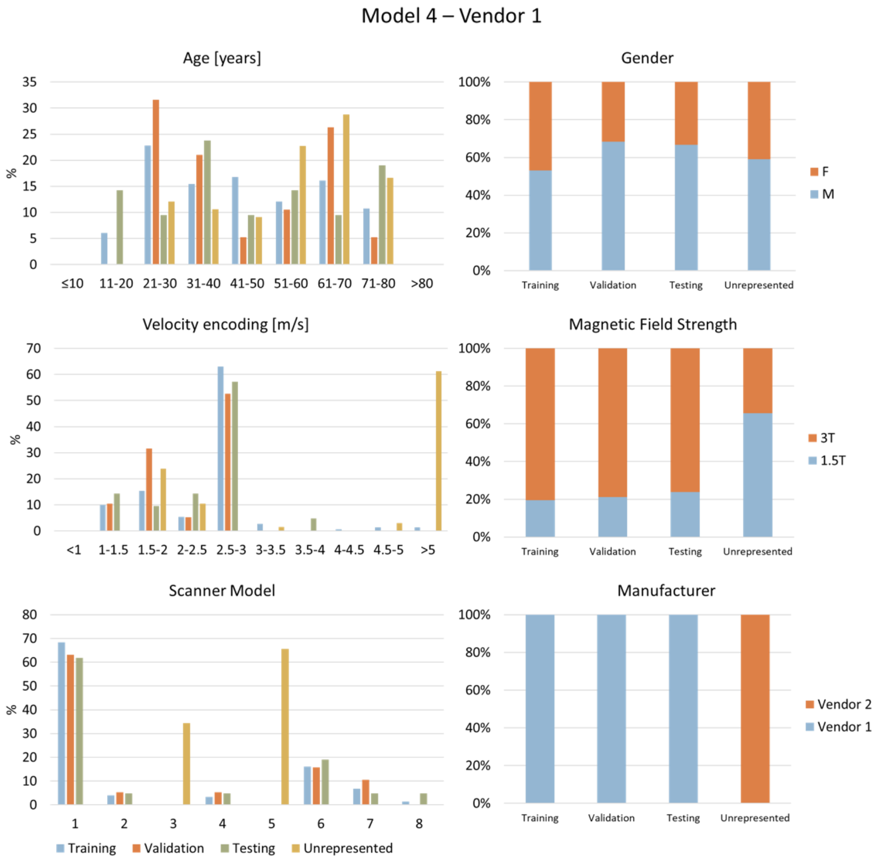

Figure 7. Model 4 (vendor 1) card. Detailed statistic information about the model-specifictraining, validation, testing and unrepresented splits.

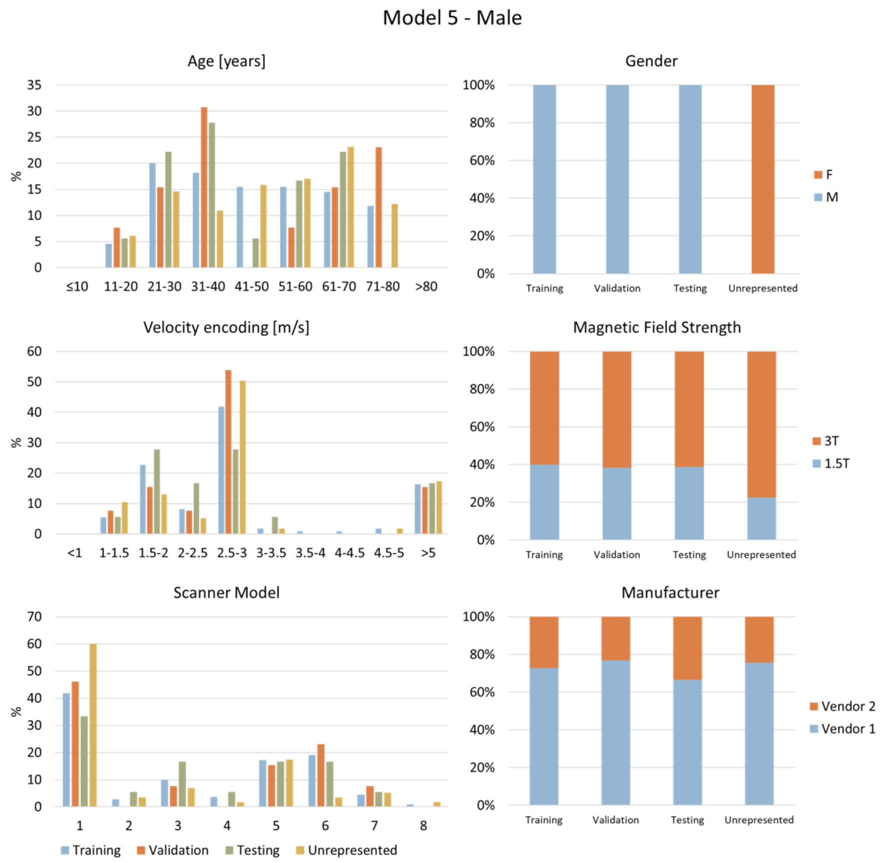

Figure 8. Model 5 (male) card. Detailed statistic information about the model-specifictraining, validation, testing and unrepresented splits.

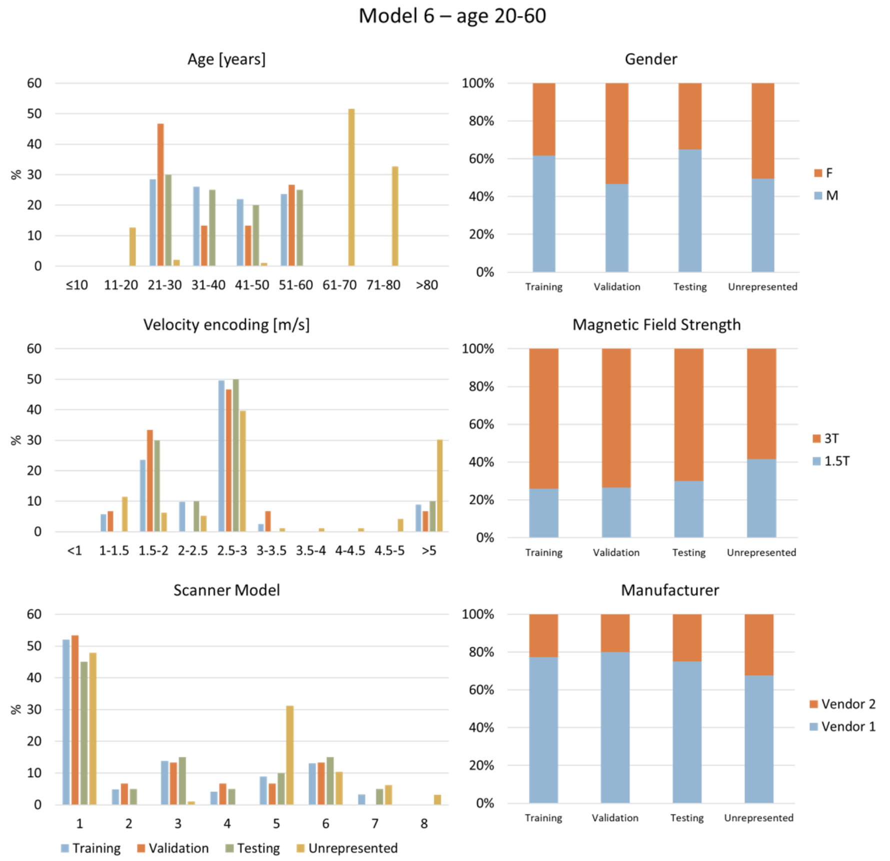

Figure 9. Model 6 (age 20-60) card. Detailed statistic information about the model-specifictraining, validation, testing and unrepresented splits.

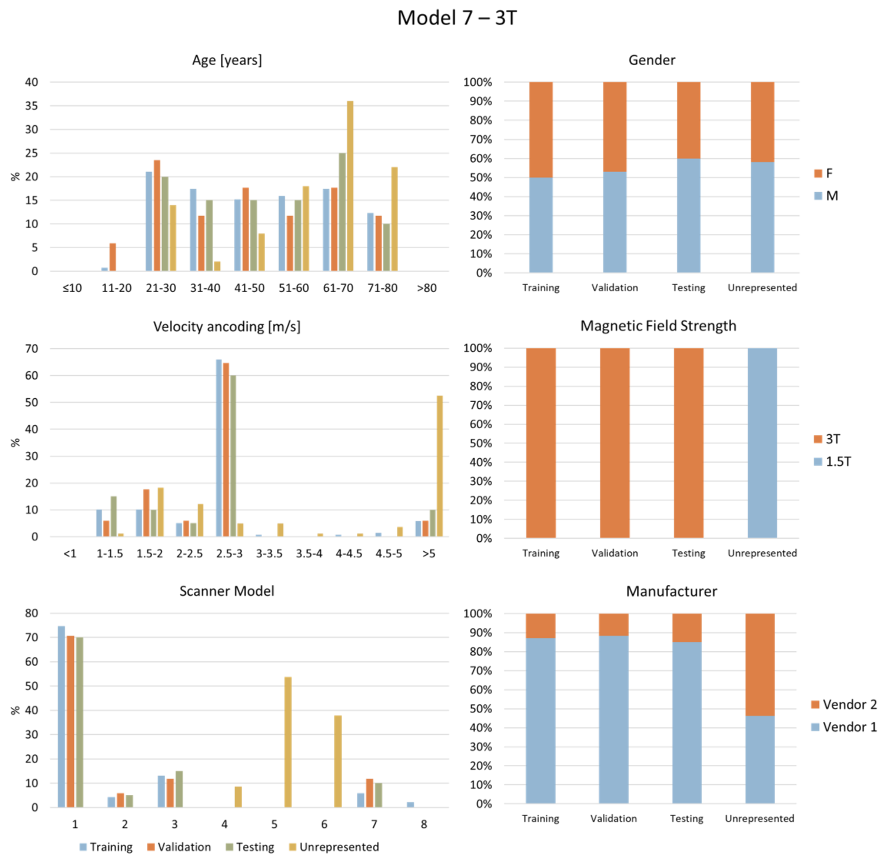

Figure 10. Model 7 (3T) card. Detailed statistic information about the model-specific training, validation, testing and unrepresented splits.
